# Quantitative myocardial perfusion SPECT/CT for the assessment of myocardial tracer uptake in patients with three-vessel coronary artery disease: Initial experiences and results

**DOI:** 10.1007/s12350-021-02735-2

**Published:** 2021-08-02

**Authors:** Sebastian Lehner, Isabel Nowak, Mathias Zacherl, Julia Brosch-Lenz, Maximilian Fischer, Harun Ilhan, Johannes Rübenthaler, Astrid Gosewisch, Peter Bartenstein, Andrei Todica

**Affiliations:** 1 Department of Nuclear Medicine, University Hospital, Ludwig-Maximilians-Universität, Munich, Germany; 2 Ambulatory Health Care Center Dr. Neumaier & Colleagues, Radiology, Nuclear Medicine, Radiation Therapy, Bahnhofstraße 24, 93047 Regensburg, Germany; 3 Department of Internal Medicine, Cardiology, University Hospital, Ludwig-Maximilians-Universität, Munich, Germany; 4 Department of Radiology, Ludwig-Maximilians-University, Munich, Germany

**Keywords:** Myocardial perfusion scintigraphy, quantitative SPECT, attenuation correction, balanced ischemia, three-vessel disease

## Abstract

**Background:**

To evaluate quantitative myocardial perfusion SPECT/CT datasets for routine clinical reporting and the assessment of myocardial tracer uptake in patients with severe TVCAD.

**Methods:**

MPS scans were reconstructed as quantitative SPECT datasets using CTs from internal (SPECT/CT, Q_INT) and external (PET/CT, Q_EXT) sources for attenuation correction. TPD was calculated and compared to the TPD from non-quantitative SPECT datasets of the same patients. SUV_max_, SUV_peak_, and SUV_mean_ were compared between Q_INT and Q_EXT SPECT datasets. Global SUV_max_ and SUV_peak_ were compared between patients with and without TVCAD.

**Results:**

Quantitative reconstruction was feasible. TPD showed an excellent correlation between quantitative and non-quantitative SPECT datasets. SUV_max_, SUV_peak_, and SUV_mean_ showed an excellent correlation between Q_INT and Q_EXT SPECT datasets, though mean SUV_mean_ differed significantly between the two groups. Global SUV_max_ and SUV_peak_ were significantly reduced in patients with TVCAD.

**Conclusions:**

Absolute quantification of myocardial tracer uptake is feasible. The method seems to be robust and principally suitable for routine clinical reporting. Quantitative SPECT might become a valuable tool for the assessment of severe coronary artery disease in a setting of balanced ischemia, where potentially life-threatening conditions might otherwise go undetected.

**Supplementary Information:**

The online version contains supplementary material available at 10.1007/s12350-021-02735-2.

## Background

Myocardial perfusion scintigraphy (MPS) with ^99m^Tc-labeled agents (^99m^Tc-2-methoxyisobutylisonitrile, sestamibi; ^99m^Tc-1,2-bis[bis(2-ethoxyethyl) phosphino] ethane, tetrofosmin) has been an important mainstay in the diagnosis and risk stratification of coronary artery disease, stress-induced ischemia, and heart failure for several decades.^1^,^2^ Combined with the evaluation of myocardial metabolism by means of ^18^F-fluorodeoxyglucose positron emission tomography (FDG PET) MPS is a powerful tool to determine the presence and amount of hibernating myocardium in heart failure patients, which might serve as a predictor for the response to myocardial revascularization therapy.^3^

MPS has traditionally made use of relative quantification with the normalization of the tracer uptake to the maximum uptake in the left ventricle. Concerns arose that three-vessel coronary artery disease (TVCAD) might be misdiagnosed due to uniformly reduced tracer uptake in the supply territories of all three main coronary arteries, rendering the myocardial perfusion scintigram unremarkable.^4^ In addition to attenuation correction, the use of more sensitive detectors or gated MPS acquisitions, one possible way to ameliorate this situation is the use of absolute quantification in myocardial single-photon emission computed tomography (SPECT) and subsequently the calculation of SUVs.^4^ With the advent of SPECT/CT imaging and advanced software reconstruction algorithms, such as SUV SPECT^®^ for Hermes Hybrid Recon™ (Hermes Medical Solutions, Stockholm, Sweden), absolute quantification of SPECT datasets has become feasible and reproducible in a manner that seems to allow its application in routine clinical use.^5^

The use of quantitative SPECT datasets to calculate SUVs and even absolute regional blood flow has been proposed and partially tested for a wide variety of clinical applications, for example, imaging of cardiac amyloidosis,^6^ breast cancer imaging,^7^ bone scintigraphy^8^, and myocardial perfusion imaging.^9^

To our knowledge, no studies have been published that systematically evaluate the use of quantitative myocardial perfusion SPECT dataset for routine clinical imaging. Furthermore, no studies have employed SUVs to evaluate myocardial perfusion in patients with TVCAD.

In the present imaging study, we aimed to address the following four major aspects:

We evaluated the feasibility of creating equal quantitative myocardial perfusion SPECT datasets using commercial software and CTs from internal (SPECT/CT) and external (PET/CT) sources for attenuation correction (AC).

We investigated whether the TPD determined from the quantitative datasets was equal to the TPD determined from standard non-quantitative datasets used in routine clinical MPS.

We compared SUV_max_, SUV_peak_, and SUV_mean_ as derived from quantitative datasets with internal and external AC to determine their reproducibility.

We compared global SUVs from patients with and without TVCAD to determine differences in global tracer uptake that might signify perfusion deficits, which might otherwise go undetected in conventional, semiquantitative analysis due to balanced ischemia.

## Materials and methods

### Study population

Cohort 1 (feasibility of quantitative reconstruction, comparison of TPD and SUVs) comprised 30 patients (25 male, mean age 66 ± 9 years, mean weight 88 ± 20 kg, mean height 175 ± 7 cm, mean BMI 29 ± 6 kg/m^2^, mean injected dose 464 ± 145 MBq).

Cohort 2 (comparison of global SUVs in patients with and without TVCAD) comprised 40 patients (20 patients without TVCAD: 15 male, mean age 64 ± 10 years, mean weight 94 ± 24 kg, mean height 174 ± 6 cm, mean BMI 31 ± 8 kg/m^2^, mean injected dose 552 ± 212 MBq; 20 patients with TVCAD verified by heart catheterization: 17 male, mean age 64 ± 11 years, mean weight 88 ± 23 kg, mean height 175 ± 7 cm, BMI 29 ± 8 kg/m^2^, mean injected dose 517 ± 157 MBq; there were no significant differences between the groups with regard to gender, age, weight, height, and injected dose).

Patients from both groups were referred to our ward for myocardial viability imaging between June 2010 and December 2016. The standard protocol at out institution usually comprises a rest myocardial perfusion SPECT as well as an 18F-FDG PET/CT scan.

Routine MPS was performed in resting conditions using ^99m^Tc-tetrofosmin on an integrated SPECT/CT scanner (Symbia, Siemens Medical Systems, Erlangen, Germany). After MPS an ^18^F-FDG PET/CT scan was performed on a dedicated PET/CT system (Biograph 64, Siemens Medical Systems, Erlangen, Germany). Patients from Cohort 2 received prior heart catheterization so that information about the state of the coronary arteries (single- or multi-vessel disease) was available. TVCAD was assumed, when all three coronary arteries (left anterior descending artery, left circumflex artery, right coronary artery) were affected by at least one stenosis of > 50% of the vessel diameter as assessed by invasive coronary angiography.

The study was conducted in accordance with the local ethics committee (Ethikkommission der LMU München).

### SPECT/CT imaging

Patients from both groups received MPS at rest. ^99m^Tc-tetrofosmin was administered intravenously. SPECT/CT scans were started 30-45 minutes after tracer injection. A dual-head hybrid SPECT/CT camera (Symbia, Siemens Medical Systems, Erlangen, Germany) was employed with a low-energy, high-resolution parallel-hole collimator. A symmetrical 20% energy window was centered at an energy level of 140 keV, the two detector heads were positioned at an angle of 90°. A 180° arc was covered by the two detector heads, 64 rotational steps were performed, each rotational projection lasting 23 seconds.

The SPECT scan was followed by a low-dose spiral CT during free breathing and without ECG gating for attenuation correction (130 keV, 20 mAs, CTDI 2.2 mGy, DLP 40 mGy*cm, 512 × 512 pixel matrix at a slice thickness of 5 mm).

### SPECT/CT scanner calibration

Scanner calibration was performed, using a uniform Jaszczak phantom (Data Spectrum Corporation, Durham, NC, USA). No inserts were used. The phantom was filled with water and 313 MBq of ^99m^Tc-pertechnetate. A conversion factor (.101 kBq/cps) was calculated by dividing the known activity by the reconstructed counts within a volume of interest defined inside the phantom.^10^

### PET/CT imaging

For the current study, only the CT component of the PET/CT scans was used for CT-based attenuation correction of the MPS datasets. The parameters of the CT scan were as follows: 120 keV, 11 mAs, CTDI .74 mGy, DLP 22 mGy*cm, and 512 × 512 pixel matrix at a slice thickness of 2 mm.

### Image reconstruction

Iterative reconstruction was performed using the Hybrid Recon Cardiology Software (Hermes Medical Solutions, Stockholm, Sweden).

The SPECT images were reconstructed in a standard, non-quantitative manner using the CT of the SPECT/CT scan (AC) and the following parameters: matrix size 64 x 64 pixels, OSEM, resolution recovery, CT-based attenuation correction, Monte Carlo-based scatter correction, 3 iterations, 16 subsets, and a FWHM post-reconstruction filter (.9 cm) was applied. SPECT and CT images were superimposed and registered in the software. Attenuation map registration was done using rigid-body translation, no rotations were permitted. Proper alignment of the datasets was evaluated visually and corrected manually, if necessary, to avoid the introduction of imaging artifacts due to misregistration.

This resulted in SPECT datasets with a slice thickness of 2.2 mm and no overlap (scaling factor approximately 2.2 mm/pixel, slice thickness 1 pixel, center-center separation 1 pixel).

For reconstruction of the quantitative datasets, the SUV SPECT^®^ plugin for Hermes Hybrid Recon™ (Hermes Medical Solutions, Stockholm, Sweden) was used in conjunction with CT datasets from either the SPECT/CT scan (Q_INT) or the PET/CT scan (Q_EXT) and the following parameters: matrix size 64 × 64 pixels, OSEM, resolution recovery, CT-based attenuation correction, Monte Carlo-based scatter correction, 4 iterations, 16 subsets, and a Gaussian post-filter (1.10 cm) was applied. Registration of the SPECT and CT datasets was carried out as described above. Again, this resulted in SPECT datasets with a slice thickness of 2.2 mm and no overlap (scaling factor approximately 2.2 mm/pixel, slice thickness 1 pixel, center-center separation 1 pixel).

As has been described before, the injected dose (corrected for decay) as well as patient-specific parameters such as height and weight were inputted and the counts per voxel were transformed into activity per volume and subsequently displayed as SUV.^11^

### Image analysis

The newly reconstructed quantitative datasets were visually compared to the standard non-quantitative datasets. We evaluated, if any obvious new artifacts from attenuation correction or image reconstruction had been introduced, such as defects or extracardiac activity. Furthermore, we visually assessed if gross image quality was comparable.

As described by Beanlands *et al* the resulting SPECT images were analyzed using the commercial software QPS with the QPET-Plugin (Cedars-Sinai, Los Angeles) to calculate the extent of the TPD^12^: after the creation of polar maps, the perfusion tracer uptake is quantified relative to the maximum tracer uptake in the polar map. The patient’s polar map is then compared to the average polar map of an integrated normative database and the TPD is calculated by the software as a combination of the extent of the perfusion deficit (percentage of the left ventricular surface area) and the severity of the perfusion deficit (reduction of the perfusion in standard deviations below the normal threshold).^13^

SUVs were analyzed using Hermes Hybrid Viewer (Hermes Medical Solutions, Stockholm, Sweden). To determine global SUV_max_, SUV_mean_, and SUV_peak_ an approximate VOI was drawn around the left ventricle to exclude extracardiac activity, then the lower threshold was set to 35% to optimally delineate the left ventricle. Delineation was controlled visually and adjusted manually, if necessary. SUV_max_ was defined as the SUV of the hottest voxel within the VOI, SUV_mean_ was defined as the average SUV of all the voxels within the VOI and SUV_peak_ was defined as the average SUV in a cubic 1 cm^3^ VOI around the area of maximum tracer uptake within the main VOI.^10^

### Statistical analysis

All variables are reported as mean ± standard deviation (SD).

Statistics were calculated with the commercial statistics software Wizard 2 (Version 2.0.4 (250), Evan Miller) and with IBM SPSS Statistics (Version 28.0.0.0 (190)).

The Shapiro-Wilk test was used to test for normal distribution.

A two-sided one-sample Student’s t-test was used to assess, if a mean was different from 0.

To compare qualitative variables between two groups, the Chi-Square test was used.

To compare quantitative variables that were not normally distributed between two groups, the Mann-Whitney *U*-Test was used.

For quantitative variables that were normally distributed, the Student’s *t* test (for dependent or independent samples) was used to compare two groups.

ANOVA adjusted for multiple comparisons with the Šidák correction was used, when more than two groups were compared.

Pearson’s *r* was calculated as a measure of linear correlation between two datasets, scatter diagrams and Bland-Altman plots were used for visualization. Furthermore, coefficients of variation were calculated for comparison of the variability of datasets, as well as intraclass correlation coefficients to assess repeatability.

An ROC (receiver operating characteristic) analysis was performed to estimate cut-off values for SUV_peak_ and SUV_max_ to differentiate between patients with and without TVCAD based on Youden’s J statistic to optimize for sensitivity and specificity.

*P* values < .05 were considered statistically significant.

## Results

### Feasibility of quantitative reconstruction and comparison of TPD

Quantitative SPECT reconstructions were feasible in all cases. Visual review of the resulting quantitative and the standard non-quantitative SPECT images revealed no discernable differences with regard to image quality and image artifacts.

**Figure 1:**
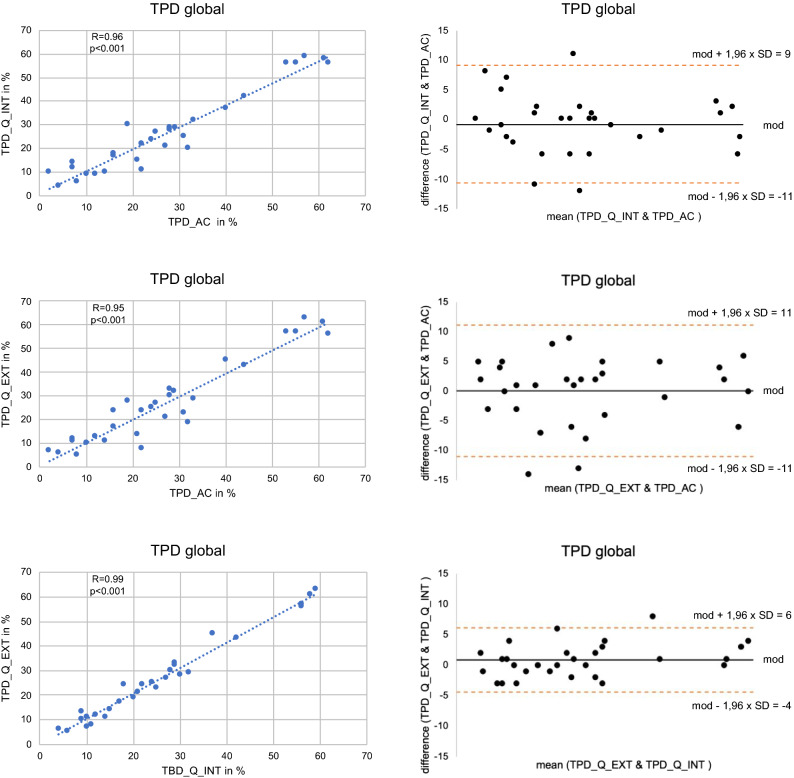
Excellent correlation between the global TPDs as determined with the three different reconstruction methods. The methods showed good agreement in the Bland-Altman plots, however, some variability was present. *mod*, mean of differences

The mean TPD showed no significant differences between the groups (TPD_AC vs TPD_Q_INT, 27 ± 17% vs 26 ± 17%; TPD_AC vs TPD_Q_EXT, 27 ±17% vs 27 ± 18%; TPD_Q_INT vs TPC_Q_EXT, 26 ± 17% vs 27 ± 18%; *P* = ns in all groups). The coefficient of variation was similar for all three methods (TPD_AC: .63; TPD_Q_INT: .65; TPD_Q_EXT: .67). Mean paired differences were − .77 ± 5.0% (TPD_Q_INT − TPD_AC), .07 ± 5.7% (TPD_Q_EXT − TPD_AC), and .83 ± 2.7% (TPD_Q_EXT − TPD_Q_INT). The mean paired differences were not significantly different from each other as well as from 0 (*P* = ns in all). The global TPDs showed an excellent correlation as well as good agreement in the Bland-Altman plots, however, some variability was present (Figure 1). The intraclass correlation coefficient for TPD_AC and TPD_Q_INT was .957, the intraclass correlation coefficient for TPD_AC and TPD_Q_EXT was .949, indicating excellent repeatability in both cases.^14^,^15^

### Comparison of global SUV_max_, SUV_peak_, and SUV_mean_

**Figure 2:**
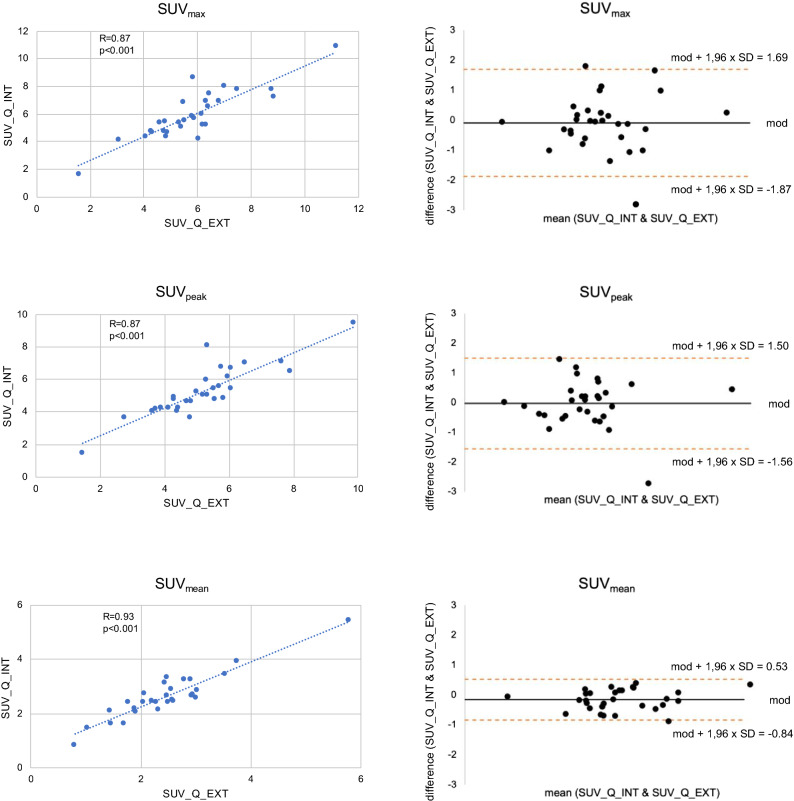
Excellent correlation between the global SUVs and good agreement in the Bland-Altman plots, however, some variability was present. *mod*, mean of differences

As shown in Table 1, mean SUV_max_ and SUV_peak_ showed no significant differences between the Q_INT and Q_EXT groups. Mean SUV_mean_, however, differed significantly between the two groups. The coefficients of variation of the parameters were similar for both methods (SUV_max_ Q_INT: .31 and SUV_max_ Q_EXT: .30; SUV_peak_ Q_INT: .30 and SUV_peak_ Q_EXT: .29; SUV_mean_ Q_INT: .37 and SUV_mean_ Q_EXT: .31). Mean paired differences were − .089 ± .91 (SUV_max_ Q_INT − Q_EXT), − .027 ± .78 (SUV_peak_ Q_INT − Q_EXT), and − .16 ± .35 (SUV_mean_ Q_INT − Q_EXT). The mean paired differences for SUV_max_ and SUV_peak_ were not significantly different from 0, the mean paired difference for SUV_mean_ was significantly different from 0 (*P* = .019). All SUVs showed an excellent correlation as well as good agreement in the Bland-Altman plots, however, some variability was present (Figure 2). The intraclass correlation coefficient for SUV_max_ Q_INT and Q_EXT was .87, for SUV_peak_ Q_INT and Q_EXT .87, and for SUVmean Q_INT and Q_EXT .92, indicating good to excellent repeatability.^14^,^15^

**Table 1:** Comparison of SUV_max_, SUV_peak_, and SUV_mean_ between Q_INT and Q_EXT groups as well as mean paired differences and SD

	Q_INT	Q_EXT	*P*	Q_INT − Q_EXT
SUV_max_	5.80 ± 1.76	5.89 ± 1.72	.599	− .089 ± .91
SUV_peak_	5.21 ± 1.54	5.24 ± 1.50	.849	− .027 ± .78
SUV_mean_	2.47 ± .90	2.63 ± .81	.031	− .16 ± .35

### Comparison of global SUV_max_ and SUV_peak_ in patients with and without TVCAD

**Figure 3:**
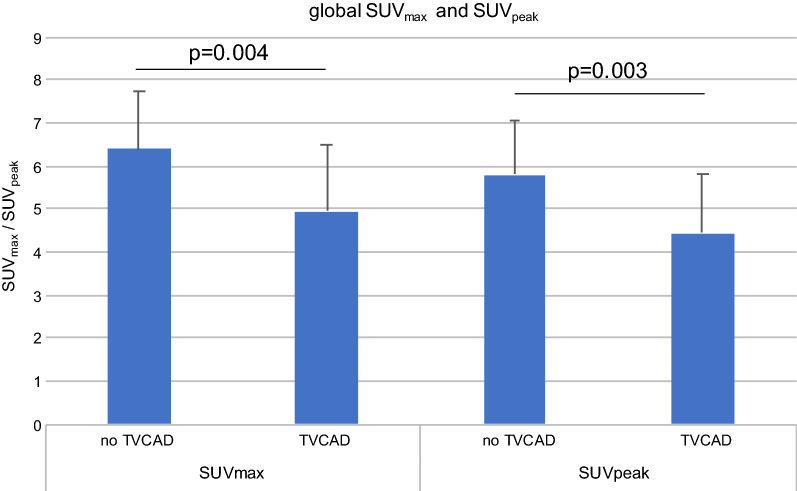
Patients with TVCAD present showed significantly reduced SUV_max_ and SUV_peak_ as compared to patients with no TVCAD present

Patients with TVCAD showed a significantly reduced SUV_max_ as compared to patients with no TVCAD present (TVCAD: SUV_max_ 4.96 ± 1.54 vs. no TVCADD: SUV_max_ 6.39 ± 1.34, *P* = .004). Likewise, patients with TVCAD showed a significantly reduced SUV_peak_ as compared to patients with no TVCAD present (TVCAD: SUV_peak_ 4.44 ± 1.40 vs no TVCAD: SUV_peak_ 5.81 ± 1.24, *P* = .003) (Figure 3).

**Figure 4:**
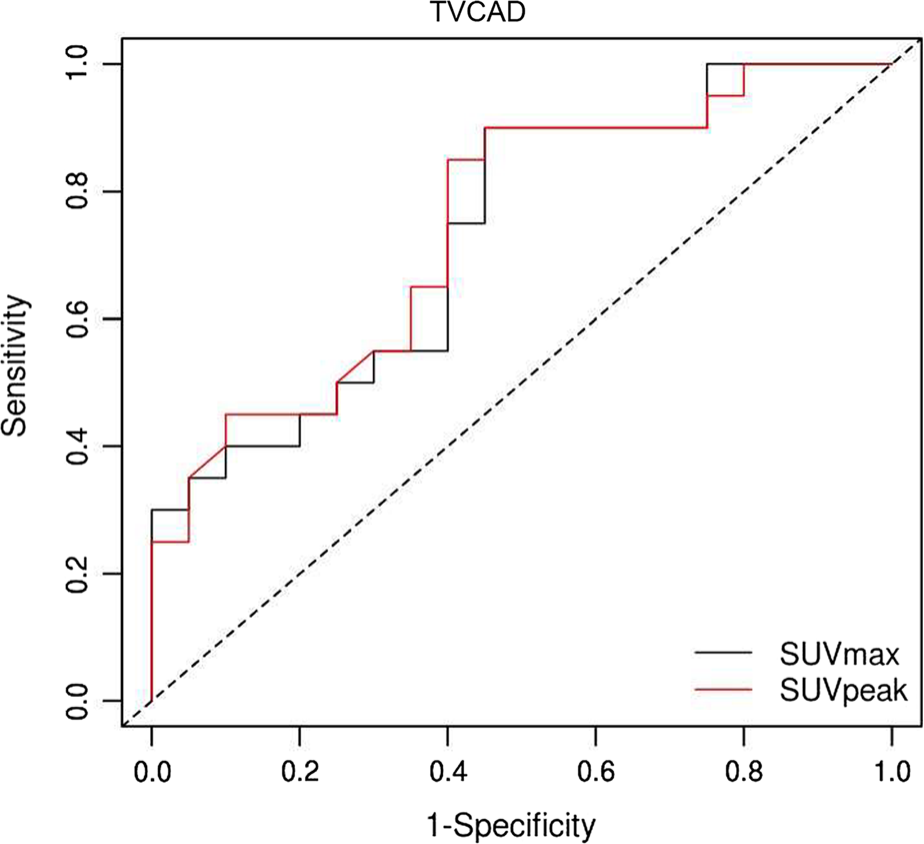
ROC analysis for SUV_peak_ and SUV_max_ to differentiate between patients with and without TVCAD, AUC = .75 for SUV_peak_ and AUC=.73 for SUV_max_. Cut-off values optimized for sensitivity and specificity are 4.62 for SUV_peak_ (sensitivity 90%, specificity 55%) and 5.04 for SUV_max_ (sensitivity 90% and specificity 55%)

ROC analysis (Figure 4) revealed a satisfactory discrimination of SUV_peak_ and SUV_max_ between patients with and without TVCAD (AUC = .75 for SUV_peak_, AUC = .73 for SUV_max_). The optimized cut-off values were 4.62 for SUV_peak_ (sensitivity 90%, specificity 55%) and 5.04 for SUV_max_ (sensitivity 90%, specificity 55%).

## Discussion

Reconstruction of quantitative SPECT datasets was feasible in all cases without any tradeoff in image quality. The TPD—a central parameter for the routine assessment of the extent of myocardial ischemia, scarring, and hibernation—could be determined from quantitative SPECT datasets (irrespective of internal or external AC) and showed a good agreement with the TPD calculated from standard non-quantitative datasets. Hence, we infer that quantitative datasets are principally suitable for routine semiquantitative image analysis. Furthermore, we could demonstrate that SUV_max_, SUV_peak_, and SUV_mean_ showed an excellent correlation and agreement between datasets reconstructed with internal and external CT sources, even though there was a statistically significant difference of the mean SUV_mean_ between groups. Patients with TVCAD showed significantly reduced global SUVs in the resting state as compared to patients without TVCAD, possibly reflecting chronically impaired perfusion in high-risk disease. Furthermore, ROC analysis suggests that SUV_peak_ and SUV_max_ show a satisfactory performance to differentiate between patients with and without TVCAD with a high sensitivity.

Visual comparison with standard non-quantitatively reconstructed datasets of the same patients did not reveal any increase in image artifacts. Image quality of the quantitative datasets was satisfactory and on par with their non-quantitative counterparts. This is in line with several other studies that evaluated quantitative SPECT in different settings and did not report any issues with the reconstructed quantitative SPECT datasets.^8^,^11^,^16^

The TPD is a central parameter in routine analysis of MPS images with the QPS Software.^17^ It represents the extent and severity of a perfusion defect and is highly correlated with the visual assessment of perfusion defects as well as summed rest, stress, and difference scores.^18^ As such, the assessment of this well-established parameter must not be impaired by new or alternative image reconstruction methods. In our study, we could demonstrate that the calculation of TPD was feasible in all cases and that the obtained values correlated well with values determined from standard non-quantitative image datasets irrespective of the CT source for AC (internal or external). Bland-Altman Plots, however, revealed that there seems to be a certain amount of variability between the different methods, especially when quantitative datasets are compared to their non-quantitative counterpart. As such, while the calculation of TPD values is certainly possible and the quantitative datasets are principally suitable for its routine evaluation, at the present time, we would suggest adhering to one method of reconstruction (quantitative or non-quantitative), especially for therapy control or follow-up examinations on a patient per patient basis, until further research into this topic is conducted.

In a next step, we were able to demonstrate that the calculated SUV_max_, SUV_peak_, and SUV_mean_ showed an excellent correlation between quantitative datasets reconstructed either with the internal CT from the SPECT/CT scan (Q_INT) or the external CT from the PET/CT scan (Q_EXT). This is especially important, since it raises confidence in the stability of the method and offers additional options for institutions with SPECT-only scanners, when external CT datasets are available. These results are in line with observations previously published by our own working group that demonstrated that attenuation correction based on internal and external CT scans for SPECT datasets was equal with regard to the quantification of perfusion deficits, scars, and hibernating myocardium.^2^ Interestingly, mean SUV_mean_ showed a small (Q_INT 2.47 ± .90 vs Q_EXT 2.63 ± .81), albeit significant difference (*P* = .031). In contrast to other SUV definitions, which rely on the hottest voxels, SUV_mean_ is dependent on the VOI size and thus on the threshold used to delineate the heart (with additional manual corrections, if required). This might subsequently lead to variations in VOI size and placement resulting in the observed significant difference, despite excellent correlation curves and good Bland-Altman plots.^19^ Bland-Altman plots revealed a certain amount of variability between the methods (Q_INT vs Q_EXT) to determine SUVs. Subsequently, as with TPD, at the present time it seems prudent to adhere to one method for repeated or follow-up examinations. Based on these results, in the subsequent parts of our study, we focused on SUV_max_ and SUV_peak_ for further evaluation of tracer uptake in patient cohorts with and without TVCAD.

Absolute quantification of myocardial tracer uptake is especially interesting in patients with TVCAD, since uniformly reduced myocardial perfusion in the territories of all three main coronary vessels—referred to as balanced ischemia—might give myocardial perfusion scintigrams normalized to the maximum tracer uptake in the myocardium an unremarkable appearance.^4^ Subsequently, a severe, potentially life-threatening condition might be misdiagnosed with possibly devastating consequences. In theory, absolute quantification of myocardial tracer uptake could have the potential to overcome this situation.^4^

Comparing global SUV_peak_ and SUV_max_ from rest myocardial perfusion scintigrams in patients with and without TVCAD, we could demonstrate that even under resting conditions global absolute myocardial tracer uptake is significantly reduced in patients with severe coronary artery disease, most likely reflecting chronically impaired myocardial perfusion. This is in line with PET-perfusion-based studies that could demonstrate that impaired myocardium (both stunned and hibernating myocardium as well as scar) showed reduced myocardial blood flow under resting conditions as compared to remote myocardium.^20^

We consider this a particularly important finding and in line with the assumption that impaired myocardial perfusion in severe coronary artery disease might be misdiagnosed in semiquantitative assessment, but revealed using SUV-based quantification, possibly leading to higher sensitivity of the examination and the detection of potentially life-threatening conditions.^4^ ROC analysis suggests that SUV_peak_ and SUV_max_ might be useful markers to differentiate between patients with and without TVCAD with a high sensitivity, which is warranted in a setting of a life-threatening disease. Specificity at 55% might have to be greatly improved, surely further research is warranted in this regard.

Furthermore, these findings might pave the way for future implementations of quantitative SPECT to assess myocardial blood flow and coronary flow reserve, bringing SPECT closer to PET, which can be considered the gold standard in this respect.^21^

## New Knowledge Gained

In our study, we could demonstrate the feasibility of absolute quantification of myocardial tracer uptake in myocardial perfusion scintigraphy. We could substantiate that the method is stable and principally suitable for routine clinical reporting from quantitative datasets, even though caution should be exerted for therapy monitoring or follow-up studies. Finally, we could show that quantitative SPECT might have the potential to become a valuable tool for the assessment of severe coronary artery disease in a setting, where potentially life-threatening conditions might otherwise go undetected.

## Limitations

Our study suffers from several limitations.

The number of patients was limited, the study population was restricted to the indication of viability testing and the patient population was dominantly male.

Due to the retrospective nature of the study, its clinical impact is limited and needs to be further elucidated by prospective investigations.

While results proved to be relatively consistent between Q_INT and Q_EXT datasets, inconsistencies arose in the evaluation of SUV_mean_. The nature of these inconsistencies is most likely attributable to somewhat differing VOI sizes, inherent in our methodological approach. Even though the absolute difference in SUV_mean_ between the two methods is so small that its clinical relevance is questionable, this seems to be proof that the method has to be further investigated and most likely refined, before routine clinical application can be considered. Additionally, further research should be conducted with regard to the properties of AC maps derived from different CT scans and their influence on SUV calculation.

When we compared TVCAD and non-TVCAD patients with regard to SUV_peak_, the non-TVCAD group contained only one patient with native coronary arteries, 9 patients had single-vessel disease and 10 patients had double-vessel disease. This was due to the nature of patient recruitment for the study. All patients were referred to our ward for viability imaging and as such usually had an extended history of CAD. The chance to find completely unaffected coronary vessels was low and these patients might not pose the optimal reference cohort. The differences in SUV_peak_ might even be more pronounced, if TVCAD patients were to be compared to a cohort of actual non-TVCAD patients with completely native coronary vessels.

Finally, within the scope of this study, only rest perfusion scans were analyzed.

## Conclusion

We could demonstrate that quantitative myocardial perfusion SPECT is able to detect reduced absolute myocardial tracer uptake in the presence of TVCAD as compared to the expected uptake in the reference cohort without TVCAD. This reduced tracer uptake might go undetected in semiquantitative assessment. At the same time, the ability for the assessment of established routine parameters is preserved in the newly reconstructed quantitative datasets. Thus, our study might pave the way for future prospective clinical investigations.

## Supplementary Information

Below is the link to the electronic supplementary material.

Supplementary file1 (M4A 1332 kb)

Supplementary file2 (PPTX 772 kb)

## Abbreviation

AC: Attenuation correction
AUC: Area under the curve
CT: Computed tomography
CTDI: Computed tomography dose index
DLP: Dose length product
FDG: ^18^F-fluorodeoxyglucose
FWHM: Full width half maximum
MPS: Myocardial perfusion scintigraphy
PET: Positron emission tomography
Q_INT: Quantitative with AC from internal CT
Q_EXT: Quantitative with AC from external CT
ROC: Receiver operating characteristic
SD: Standard deviation
SPECT: Single-photon emission computed tomography
SUV: Standardized uptake value
Tc: Technetium
TPD: Total perfusion deficit
TVCAD: Three-vessel coronary artery disease
