# Supplementary material for: Quantitative myocardial perfusion SPECT/CT for the assessment of myocardial tracer uptake in patients with three-vessel coronary artery disease: Initial experiences and results
Source: J Nucl Cardiol. 2021 Aug 2;29(5):2511–20. doi: 10.1007/s12350-021-02735-2 (PMC9553851; doi:10.1007/s12350-021-02735-2)
Supplement: Supplementary file 2 — Supplementary file2 (PPTX 772 kb) [file 12350_2021_2735_MOESM2_ESM.pptx]

## Slide 1
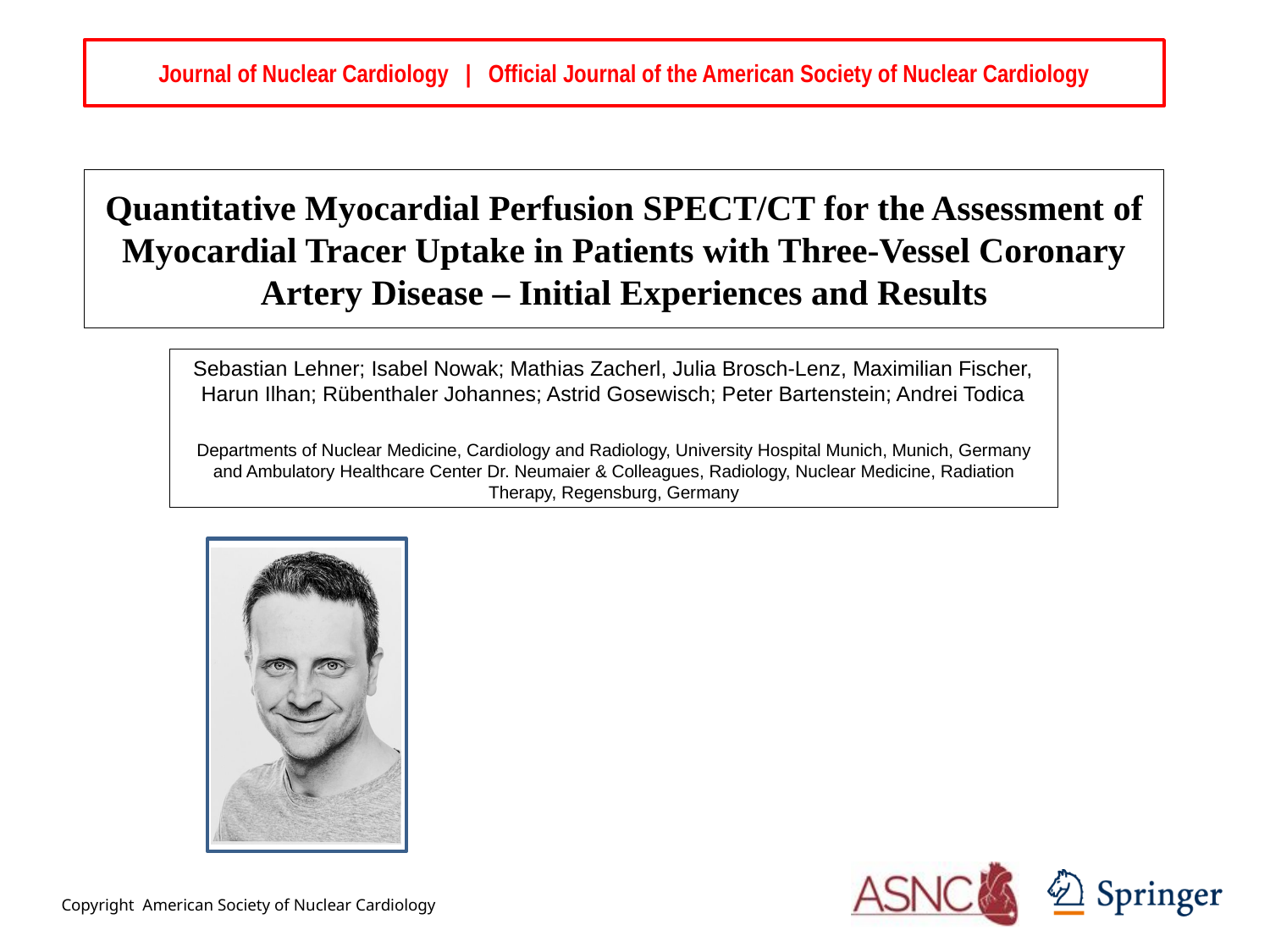

Journal of Nuclear Cardiology | Official Journal of the American Society of Nuclear Cardiology
# Quantitative Myocardial Perfusion SPECT/CT for the Assessment of Myocardial Tracer Uptake in Patients with Three-Vessel Coronary Artery Disease – Initial Experiences and Results
Sebastian Lehner; Isabel Nowak; Mathias Zacherl, Julia Brosch-Lenz, Maximilian Fischer, Harun Ilhan; Rübenthaler Johannes; Astrid Gosewisch; Peter Bartenstein; Andrei Todica
Departments of Nuclear Medicine, Cardiology and Radiology, University Hospital Munich, Munich, Germany and Ambulatory Healthcare Center Dr. Neumaier & Colleagues, Radiology, Nuclear Medicine, Radiation Therapy, Regensburg, Germany
Head shot of author
required
Copyright American Society of Nuclear Cardiology

## Slide 2
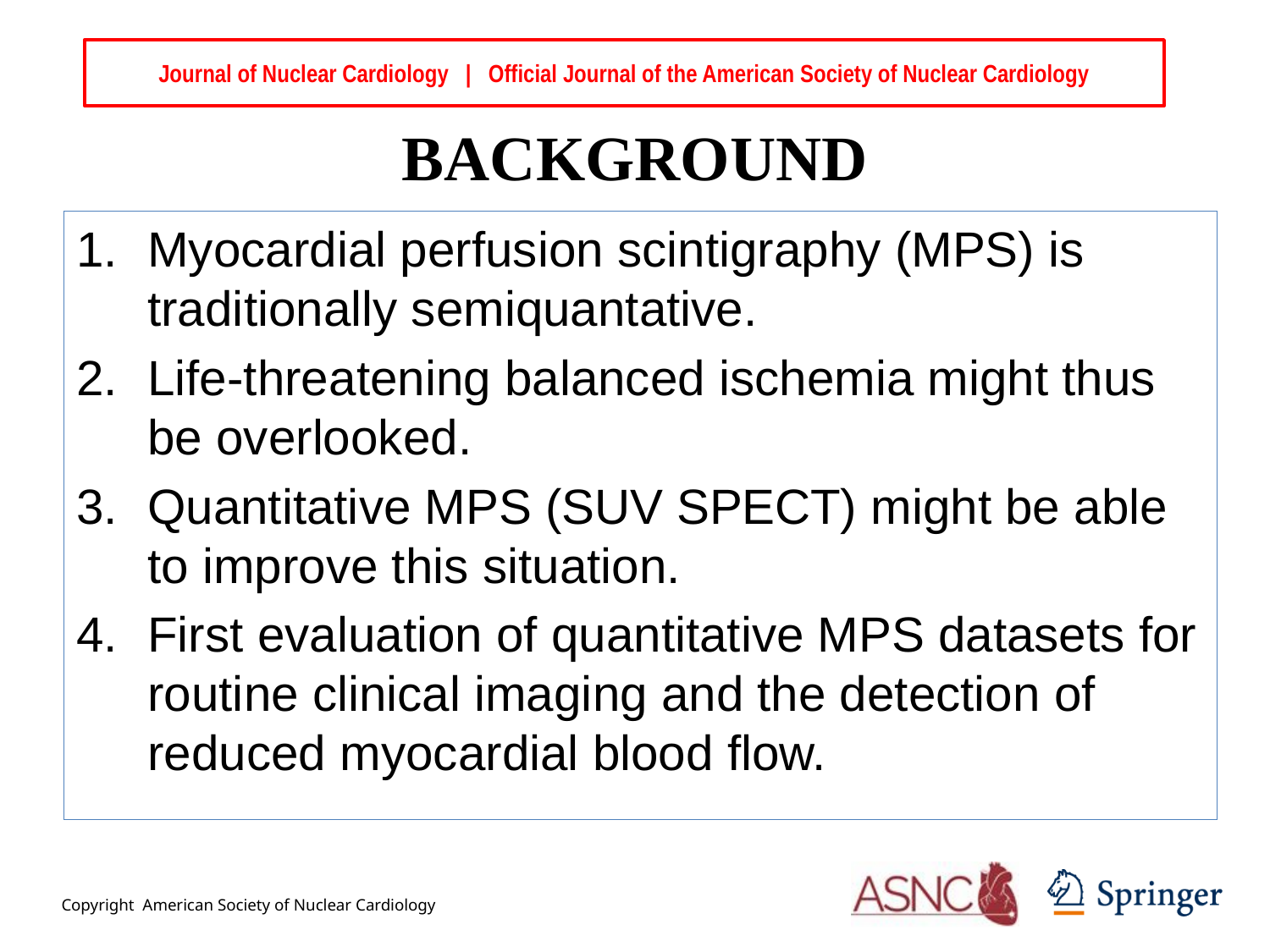

Journal of Nuclear Cardiology | Official Journal of the American Society of Nuclear Cardiology
# BACKGROUND
Myocardial perfusion scintigraphy (MPS) is traditionally semiquantative.
Life-threatening balanced ischemia might thus be overlooked.
Quantitative MPS (SUV SPECT) might be able to improve this situation.
First evaluation of quantitative MPS datasets for routine clinical imaging and the detection of reduced myocardial blood flow.
Copyright American Society of Nuclear Cardiology

## Slide 3
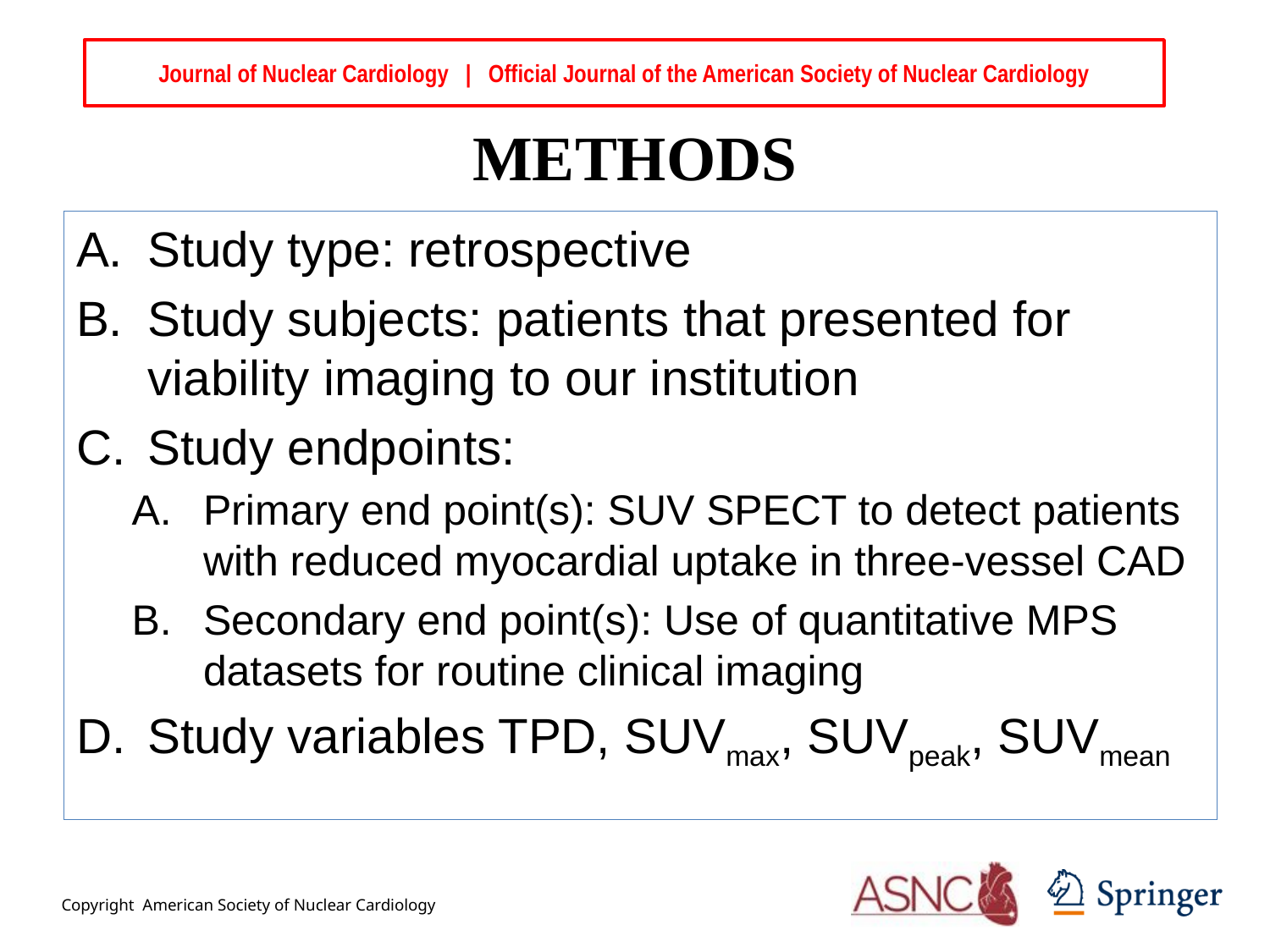

Journal of Nuclear Cardiology | Official Journal of the American Society of Nuclear Cardiology
# METHODS
Study type: retrospective
Study subjects: patients that presented for viability imaging to our institution
Study endpoints:
Primary end point(s): SUV SPECT to detect patients with reduced myocardial uptake in three-vessel CAD
Secondary end point(s): Use of quantitative MPS datasets for routine clinical imaging
Study variables TPD, SUVmax, SUVpeak, SUVmean
Copyright American Society of Nuclear Cardiology

## Slide 4
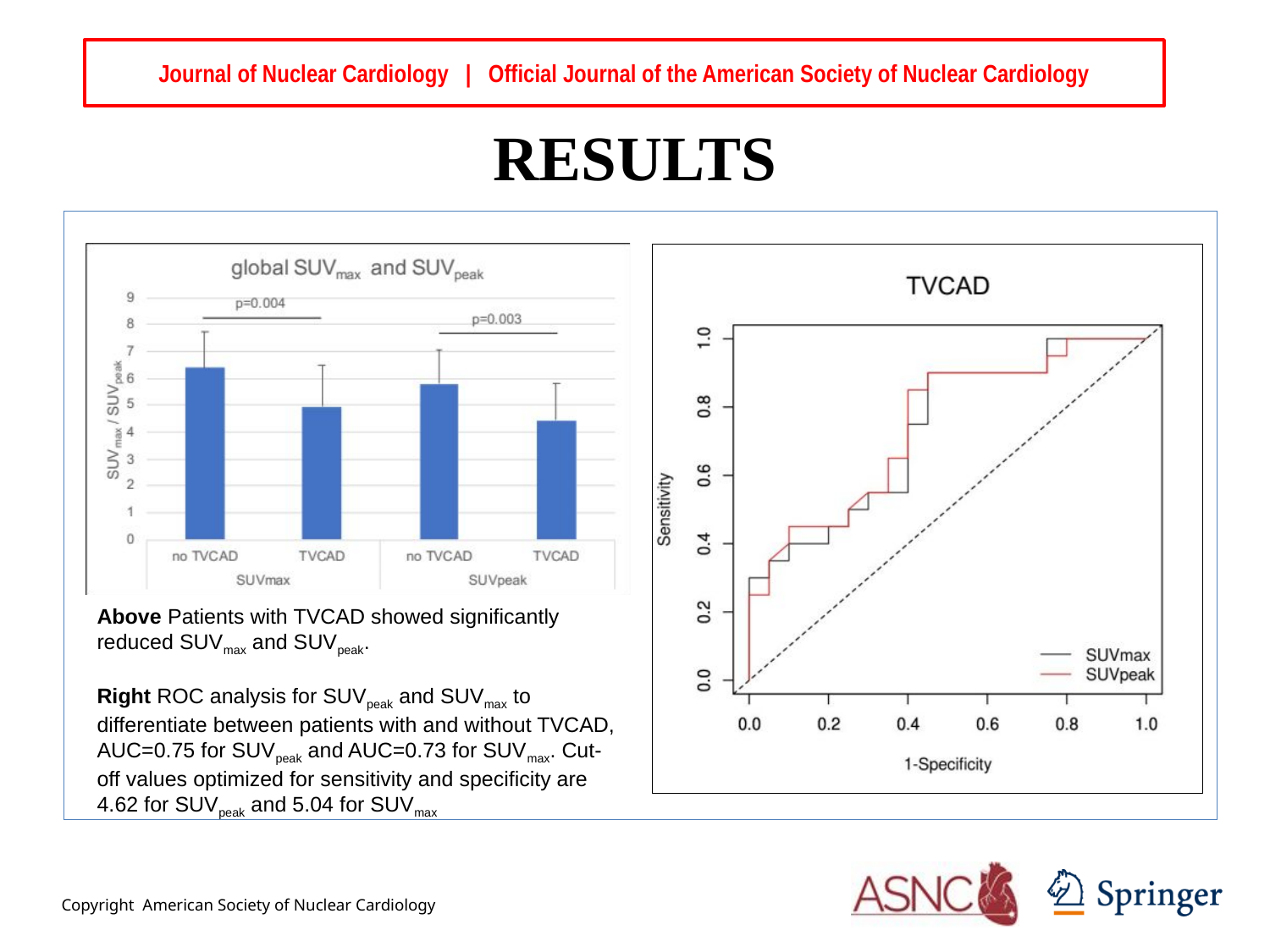

Journal of Nuclear Cardiology | Official Journal of the American Society of Nuclear Cardiology
# RESULTS
Above Patients with TVCAD showed significantly reduced SUVmax and SUVpeak.
Right ROC analysis for SUVpeak and SUVmax to differentiate between patients with and without TVCAD, AUC=0.75 for SUVpeak and AUC=0.73 for SUVmax. Cut-off values optimized for sensitivity and specificity are 4.62 for SUVpeak and 5.04 for SUVmax
Copyright American Society of Nuclear Cardiology

## Slide 5
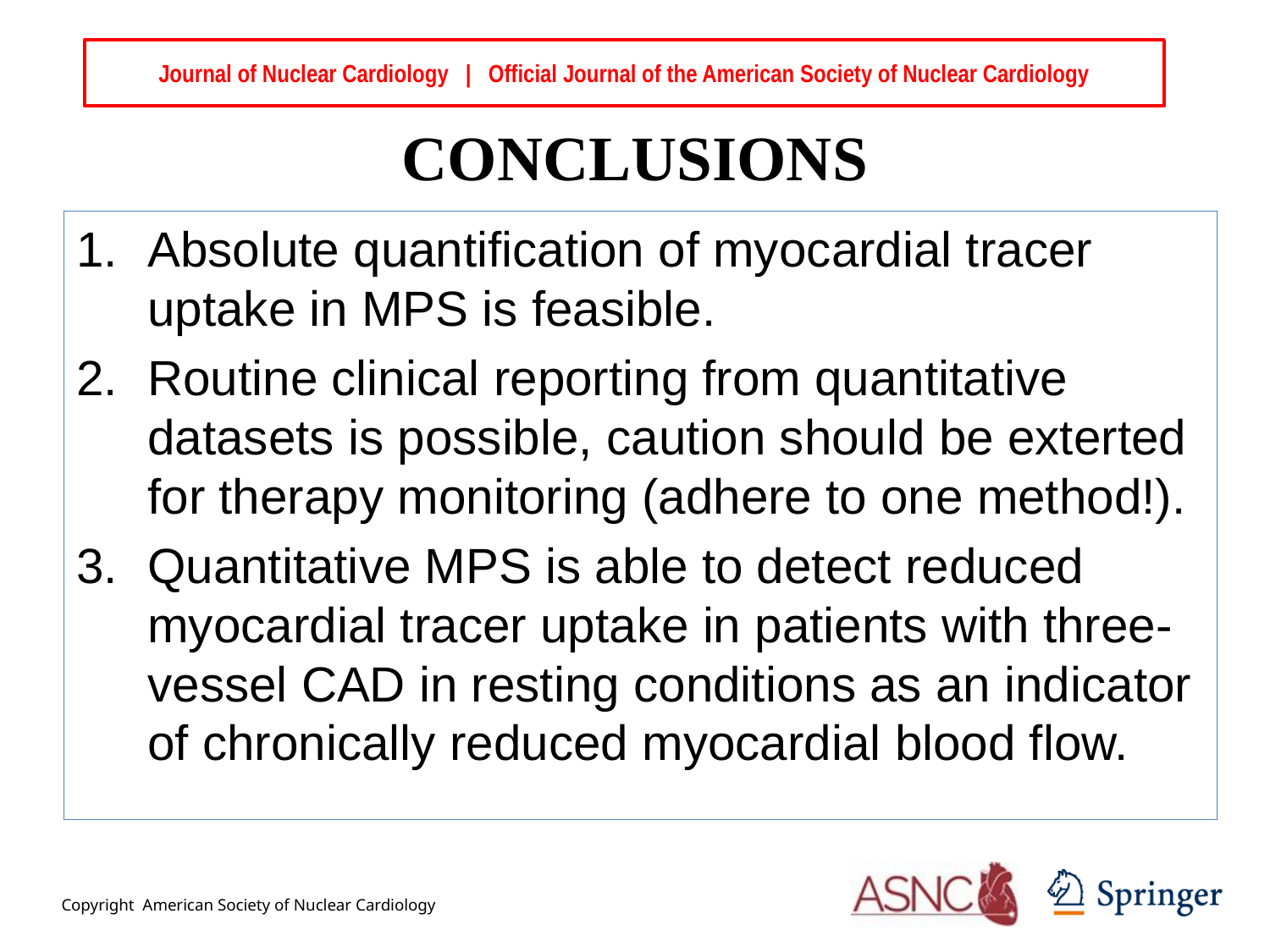

Journal of Nuclear Cardiology | Official Journal of the American Society of Nuclear Cardiology
# CONCLUSIONS
Absolute quantification of myocardial tracer uptake in MPS is feasible.
Routine clinical reporting from quantitative datasets is possible, caution should be exterted for therapy monitoring (adhere to one method!).
Quantitative MPS is able to detect reduced myocardial tracer uptake in patients with three-vessel CAD in resting conditions as an indicator of chronically reduced myocardial blood flow.
Copyright American Society of Nuclear Cardiology
